# The Chloroplastic Small Heat Shock Protein Gene *KvHSP26* Is Induced by Various Abiotic Stresses in *Kosteletzkya virginica*

**DOI:** 10.1155/2021/6652445

**Published:** 2021-02-03

**Authors:** Xiaohua Liu, Lizi Zhao, Jianzhao Li, Lijun Duan, Kai Zhang, Xuqiang Qiao, Weihuan Li, Chengchao Zheng, Xiaoli Tang, Hongxia Zhang

**Affiliations:** ^1^The Engineering Research Institute of Agriculture and Forestry, Ludong University, 186 Hongqizhong Road, Yantai, Shandong Province, China 264025; ^2^College of Agriculture, Ludong University, 186 Hongqizhong Road, Yantai, Shandong Province, China 264025; ^3^Key Laboratory of Molecular Module-Based Breeding of High Yield and Abiotic Resistant Plants in Universities of Shandong (Ludong University), Ludong University, 186 Hongqizhong Road, Yantai, Shandong Province, China 264025; ^4^Biochip Laboratory, Yantai Yu-Huang-Ding Hospital, Yantai, Shandong Province, China 26400; ^5^State Key Laboratory of Crop Biology, College of Life Sciences, Shandong Agricultural University, Tai'an, Shandong Province, China 271018

## Abstract

Small heat shock proteins (sHSPs) are a group of chaperone proteins existed in all organisms. The functions of sHSPs in heat and abiotic stress responses in many glycophyte plants have been studied. However, their possible roles in halophyte plants are still largely known. In this work, a putative *sHSP* gene *KvHSP26* was cloned from *K. virginica*. Bioinformatics analyses revealed that *KvHSP26* encoded a chloroplastic protein with the typical features of sHSPs. Amino acid sequence alignment and phylogenetic analysis demonstrated that KvHSP26 shared 30%-77% homology with other sHSPs from Arabidopsis, cotton, durian, salvia, and soybean. Quantitative real-time PCR (qPCR) assays exhibited that *KvHSP26* was constitutively expressed in different tissues such as leaves, stems, and roots, with a relatively higher expression in leaves. Furthermore, expression of *KvHSP26* was strongly induced by salt, heat, osmotic stress, and ABA in *K. virginica*. All these results suggest that *KvHSP26* encodes a new sHSP, which is involved in multiple abiotic stress responses in *K. virginica*, and it has a great potential to be used as a candidate gene for the breeding of plants with improved tolerances to various abiotic stresses.

## 1. Introduction

To cope with the adverse environmental conditions throughout their entire life cycle, plants have evolved a serial of sophisticated mechanisms [1, 2]. High temperature is one of the abiotic stresses which disturb the homeostasis of plant cells and the growth and development of plants. At this point, heat shock response (HSR), one of the most effective mechanisms to deal with temperature change, will be initiated, and many heat shock responsive genes, including heat shock proteins (HSPs), will be activated upon heat shock stress [3]. HSPs can bind directly to the denatured proteins to help their refolding and prevent their further aggregation and improve cell homeostasis under heat stress condition accordingly [4–6].

Due to their high expression under heat stress condition, HSPs function as one of the most important kinds of chaperones in archaea, prokaryote, and eukaryote [7–9]. Based on their molecular weight, five HSP protein families have been identified: HSP20s/sHSPs, HSP60s, HSP70s, HSP90s, and HSP100s [10–12]. HSP20s/sHSPs are the smallest members, with a molecular weight from 12 to 42 kDa [7]. Since the molecular weights of most sHSPs range from 15 to 22 kDa, they are also called as HSP20s [13]. Different from HSP60s, HSP70s, HSP90s, and HSP100s which are highly conserved, sHSPs are a little heterogeneous, with a slightly diversified N-terminal and a highly conservative C-terminal containing the *α*-crystallin domain (ACD)/HSP20 domain of sHSPs. Therefore, sHSPs are also referred as *α*-crystallin proteins [14, 15]. In addition to the two essential components, a signal/transfer peptide in the N-terminal and a C-terminal extension might be contained in different kinds of sHSPs, and the existence or not of these sequences reflects the subcellular localization of sHSPs [7]. Based on the subcellular localization, sHSPs are divided into eleven subfamilies: endoplasmic reticulum (ER), peroxisome (PX), chloroplast (CP), mitochondria (MTII), nucleus (CIII), mitochondria, and chloroplast colocalization (MTI/CP), as well as five cytoplasm classes (CI, CII, CIV, CV, and CVI), in angiosperms such as *Arabidopsis thaliana* [16–18]. Different localizations of sHSPs indicate that they might have different functions in cells.

Like other HSPs, sHSPs played crucial roles in plant response to both biotic and abiotic stresses. They function in the process of protein aggregation and nascent peptide assembling and bind to denatured proteins to work as chaperones together with other ATP-dependent HSP members such as HSP70 and HSP100 [14, 19–23]. In pepper, the small heat shock protein CaHSP25.9 positively regulated the tolerance to heat, salt, and drought stress [12]. Another pepper small heat shock protein CaHSP16.4 was involved in both heat and drought tolerance [24]. In *Malus sieversii*, MsHSP16.9 increased the activities of antioxidant enzymes and activated the signaling pathway in response to heat stress [23]. *AtHsp21* from Arabidopsis, *TaHSP26* from *Triticum aestivum*, and *CsHSP17.2* from *Camellia sinensis* were all found to participate in thermotolerance in plants. Meanwhile, some sHSPs could protect translation factors under heat stress condition [25–27]. In tomato, sHSPs were found to take part in the protection against chilling [28]. In *Oryza sativa*, OsHSP18 and OsHSP18.2 played important roles in both biotic and abiotic stress responses [29, 30]. In *Populus trichocarpa*, PtHsp17.8 took part in both heat and salt stress responses [31]. In *Closterium ehrenbergii*, two small heat shock proteins sHSP10 and sHSP17.1 responded to both heat and heavy metal stresses [32]. In addition, MsHSP17.7, a cytoplasmic sHSP from *Medicago sativa*, responded to high temperature, salt, drought, and oxidative stress [33].

As a typical perennial halophyte, *K. virginica* has great potential to be grown as a grain crop in saline soil [34–39]. Although the functions of sHSPs have been well studied in glycophyte plants, their possible roles in halophyte plants are still largely not clear. Previously, we performed transcriptomic gene expression analyses in *K. virginica* in responding to salt stress and found that *KvHSP26*, a small heat shock protein-encoding gene, was significantly upregulated in the salt treated plants [40]. In this work, we isolated the full-length gene of *KvHSP26* from *K. virginica* and examined its expression in response to different abiotic stress treatments. We report that *KvHSP26* encodes a chloroplastic sHSP homologous to those from other reported glycophyte plants with a ubiquitous expression pattern in *K. virginica*. We also showed that the expression of *KvHSP26* was upregulated by salt, heat, osmotic stress, and ABA. Our findings indicate that *KvHSP26* could play an important role in the tolerance of *K. virginica* to different abiotic stresses.

## 2. Materials and Methods

### 2.1. Plant Materials and Growth Conditions

*K. virginica* seeds, initially collected from the Ecomonitoring Station of YIC-CAS, Yellow River Delta, Shandong Province, China, were sterilized with 70% alcohol for 30 seconds and 2.6% sodium hypochlorite for 10 minutes and rinsed three times with sterile water. Then, the sterilized seeds were placed on MS (Murashige and Skoog) plates for germination. One-week-old seedlings were transplanted into plastic pots as described previously [41]. The growth conditions (photoperiod, humidity, and nutrition) of *K. virginica* plants were the same as described previously [39, 40].

### 2.2. Experimental Treatments and Sample Collection

To analyze the expression profiles of *KvHSP26*, leaves, stems, and roots were collected from four-week-old *K. virginica* plants. For stress treatments, four-week-old plants were watered with 1/10 Hoagland's nutrient solution supplemented with 300 mM NaCl, 15% PEG6000, or 100 *μ*M ABA for 0, 2, 6, 12, and 24 hours. For the high- and low-temperature treatments, four-week-old plants were kept at 42°C or 4°C for 0, 2, 6, 12, and 24 hours. For the treatment with different salt concentrations, four-week-old plants were watered with 1/10 Hoagland's nutrient solution supplemented with 0, 100, 200, 300, or 400 mM NaCl for 24 hours. At least six plants at the same size and growth status were used for each treatment, and three biological replicates were performed for each treatment. Samples were collected at each time point, quickly frozen in liquid nitrogen, and saved in -80°C for RNA extraction.

### 2.3. Total RNA Extraction and cDNA Synthesis

Total RNAs were extracted with the RNAprep Pure Plant Plus Kit (Polysaccharides & Polyphenolics-rich) (TIANGEN®, Beijing, China) following the manufacturer's instruction. RNA quality and concentration were detected with NanoPhotometer®-N50 (Implen, Germany). Genomic DNA was removed, and cDNA was synthesized with FastKing RT Kit (TIANGEN®, Beijing, China) following the user manual. The quality of cDNA was measured with NanoPhotometer®-N50 and used for the subsequent gene cloning and RT-qPCR analyses.

### 2.4. Isolation and Characterization Analysis of KvHSP26 Gene

Based on the core sequence reported in our previous study (accession number GCJL00000000), the full-length sequence of *KvHSP26* gene was cloned from *K. virginica* via rapid amplification of cDNA ends (RACE) using gene specific forward (F) and reverse (R) primers (Table 1). The amplified products were connected into the cloning vector (pEASY®-T1) for sequence confirmation. KvHSP26 protein amino acid sequence was predicted with Open Reading Frame Finder (https://www.ncbi.nlm.nih.gov/orffinder). Physical and chemical parameters, subcellular localization, transfer peptide (cTP) sequences, and conserved domain of KvHSP26 were analyzed with ProtParam (https://web.expasy.org/protparam), TargetP-2.0 (http://www.cbs.dtu.dk/services/ TargetP), ChloroP 1.1 (http://www.cbs.dtu.dk/services/ChloroP), and PROSITE (https://prosite.expasy.org). Multiple sequence alignment of KvHSP26 with sHSPs from other plant species was conducted with DNAMAN 7.0. Phylogenetic analysis was accomplished with MEGA 6.0 employing the neighbor-joining method with the *p*-distance model, and the number of bootstrap replication was set as 1000 [42].

### 2.5. Quantitative Real-Time PCR (qPCR)

For qPCR analyses, a total amount of 100 ng cDNA was used in each 20 *μ*L qPCR reaction system. The housekeeping genes *Kv18SrRNA* and *KvEF1-α* were used as internal controls. All the gene-specific primers designed with Primer Premier 5.0 are listed in Table 1. qRCR reactions were performed with the ABI StepOnePlus™ Real-Time PCR Instrument (Applied Biosystems by Thermo Fisher Scientific) and the SuperReal PreMix (SYBR Green) (TIANGEN®, Beijing, China). The components for each qPCR reaction (20 *μ*L) were: 10 *μ*L 2x SuperReal PreMix Plus, 0.6 *μ*L forward primer, 0.6 *μ*L reverse primer, 2 *μ*L cDNA template, 2 *μ*L ROX Reference Dye, and 4.8 *μ*L ddH_2_O. The procedures were 95°C initial denaturation 15 min, 40 cycle of 95°C denaturation 10 s, and 60°C annealing 30 s. The melting curve was obtained by heating the sample from 60°C to 95°C. Every sample was measured in triplicate to guarantee the accuracy.

### 2.6. Statistical Analysis

The relative expression levels of genes were all conducted and analyzed with the 2^−*ΔΔ*Ct^ method. Data were analyzed with SPSS Statistics 20, and all the figures were plotted with SigmaPlot 12.5. At least three biological replicates were performed for every experiment.

## 3. Results

### 3.1. KvHSP26 Encodes a Chloroplast Small Heat Shock Protein

**Figure 1:**
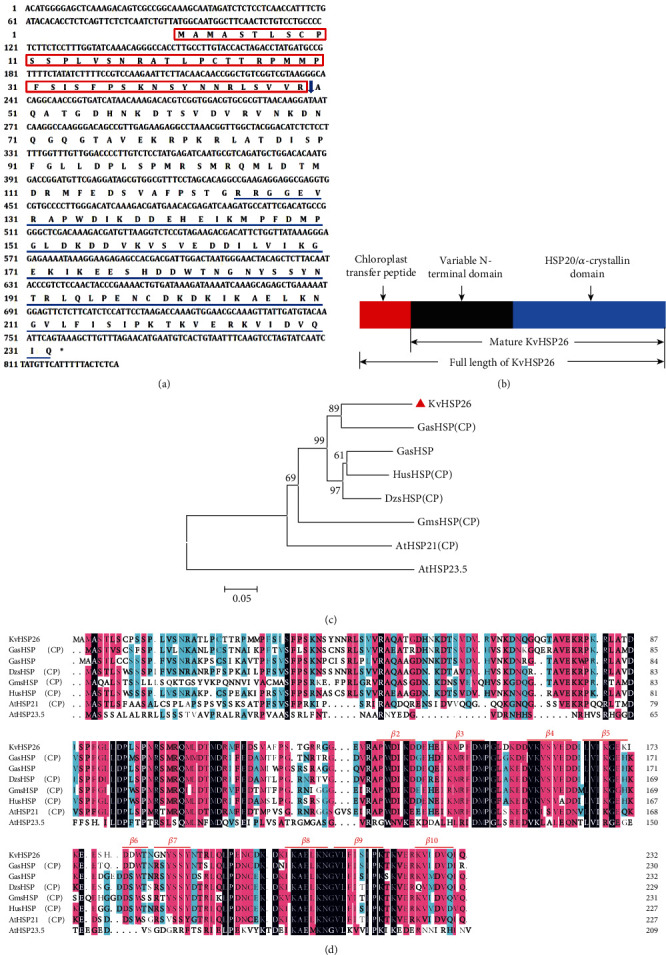
Sequence, conserved domain, multiple sequence alignment, and phylogenetic analyses of KvHSP26. (a) The full-length nucleotide and predicted amino acid sequences of *KvHspP26* gene. Each amino acid and its corresponding sequence to its encoding codon were listed. The amino acid sequence corresponding to the chloroplast transfer peptide was shown in a red frame, and the splice site was indicated with a solid blue arrow. The amino acid sequence standing for the conserved HSP20 domain in the C-terminal was underlined in blue. (b) Predicted KvHSP26 protein. (c) Phylogenetic tree of KvHSP26 (MT820948) and sHSPs from other plant species. GasHSP (CP): chloroplastic-like small heat shock protein of *Gossypium arboretum* (NP_001316945.1); GasHSP: small heat shock protein of *Gossypium arboretum* (KAA3470224.1); HusHSP (CP), chloroplastic small heat shock protein of *Herrania umbratica* (XP_021290181.1); DzsHSP (CP): chloroplastic small heat shock protein of *Durio zibethinus* (XP_022734027.1); GmsHSP (CP), chloroplastic small heat shock protein of *Glycine max* (NP_001347237.1); AtHSP21 (CP): heat shock protein 21 of *Arabidopsis thaliana* (NP_194497.1); AtHSP23.5: heat shock protein 23.5 of *Arabidopsis thaliana* (NP_199957.1). (d) Amino acid sequence alignment of KvHSP26 with its orthologous and paralogous sHSPs from other plant species.

To understand the possible functions of *sHSPs* in halophyte plants, the full-length nucleotide sequence of the *KvHSP26* gene, including 90 bp 5′-untranslated region (UTR), 71 bp 3′-UTR, and 699 bp Open Reading Frame (ORF), was cloned from *K. virginica* (GenBank accession number MT820948). *KvHSP26* encoded a 26.09 kDa protein consisting of 232 amino acids with a theoretical isoelectric point of 6.34 (Figure 1(a)). Therefore, the protein was named as KvHSP26. Aliphatic index analysis of KvHSP26 indicated that it possessed a value of as high as 70.95, a typical property of heat stable proteins. Further gene structure analyses revealed that KvHSP26 contained a C-terminal sHSP domain from amino acids 125 to 231, a chloroplast transfer peptide from amino acids 1 to 49, and a cleavage site between amino acids 49 and 50, suggesting that it is a chloroplast (CP) sHSP protein (Figure 1(a)). Compared with previous reports on the structure and conserved domain of sHSPs, a structure model of KvHSP26 containing a chloroplast transfer peptide (red), a variable N-terminal (black), and a conservative C-terminal HSP20 domain (blue) was shown (Figure 1(b)). Once the chloroplast transfer peptide was cut off at the cleavage site, the mature KvHSP26 protein would be transferred into the chloroplast. Therefore, the mature KvHSP26 was different from the full-length KvHSP26 (Figure 1(b)).

A phylogenetic tree of KvHSP26 and sHSPs from other plant species was generated, in which KvHSP26 exhibited the closest relationship to GasHSP from *Gossypium arboretum* (Figure 1(c)). Further BLAST search analysis of KvHSP26 with reference of NCBI database (https://blast.ncbi.nlm.nih.gov/Blast.cgi) revealed that KvHSP26 shared 30-77% homology with sHSPs from other plant species such as Arabidopsis, soybean, cotton, and durian (Figure 1(d)). All of them possessed nine conserved *β*-sheets (*β*2-*β*10) in the conserved C-terminal and variable N-terminal.

### 3.2. Expression of KvHSP26 Is Induced by Salt Stress

**Figure 2:**
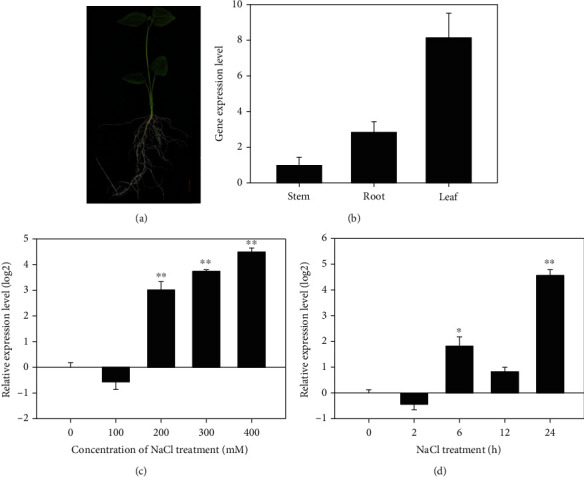
*KvHSP26* expression analyses: (a) a representative of four-week-old *K. virginica* plants used in this research; (b) relative expression of *KvHSP26* in the roots, stems, and leaves of four-week-old *K. virginica* plants; (c) relative expression of *KvHSP26* in response to different concentrations of NaCl treatments; (d) relative expression of *KvHSP26* after being treated with 300 mM NaCl for 0, 2, 6, 12, and 24 hours. The results were the means of three biological replicates. The error bars were the standard deviations of the three replicates. ∗ and ∗∗ stand for the significant differences at *p* < 0.05 and *p* < 0.01, respectively.

To know whether the expression of *KvHSP26* was affected by abiotic stress in *K. virginica*, RT-qPCR analyses were performed with one-month-old plants treated with high salt stress (Figure 2(a)). Under normal growth condition, *KvHSP26* was ubiquitously expressed in all the tested tissues of *K. virginica*, with a relatively higher expression in leaves than in roots and stems (Figure 2(b)). When the plants were subjected to salt stress, the expression of *KvHSP26* was upregulated (Figure 2(c)). Upon treatments with 200 mM to 400 mM NaCl, the expression level of *KvHSP26* continuously increased. When treated with 300 mM NaCl for different time periods, the expression level of *KvHSP26* also increased after 6 hours, slightly decreased after 12 hours, and then dramatically increased after 24 hours (Figure 2(d)).

### 3.3. Expression of KvHSP26 Is Upregulated by Heat, Osmotic Stress, and ABA

**Figure 3:**
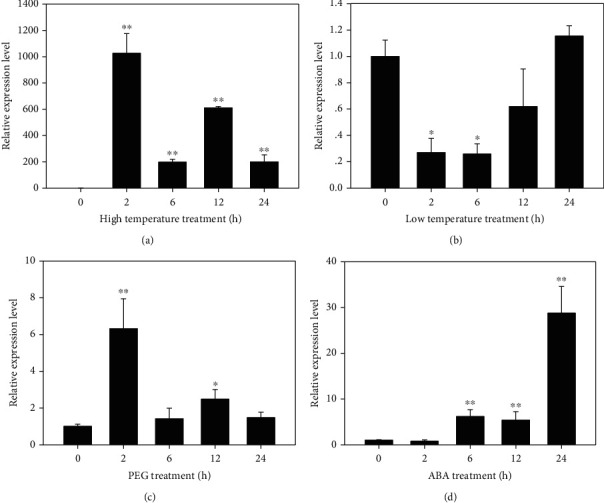
Expression analyses of *KvHSP26* in response to abiotic stress and ABA treatments. Four-week-old *K. virginica* plants were treated at 42°C or 4°C or with 15% PEG6000 or 100 *μ*M ABA for 0, 2, 6, 12, and 24 hours. (a, b) Relative expression levels of *KvHSP26* in response to high (42°C) and low (4°C) temperature treatments. (c, d) Relative expression levels of *KvHSP26* in response to 15% PEG6000 and 100 *μ*M ABA treatments. The results were the means of three biological replicates. The error bars were the standard deviations of three replicates. ∗ and ∗∗ stand for the significant differences at *p* < 0.05 and *p* < 0.01, respectively.

To further understand whether the expression of *KvHSP26* is also regulated by other abiotic stresses like other reported sHSPs, we investigated its transcript levels in response to heat, low temperature, osmotic stress, and ABA treatments. Similar to the salt stress treatment, the expression of *KvHSP26* was upregulated by high temperature, PEG, and ABA treatments (Figures 3(a)–3(d)). Upon treatments with high temperature and 15% PEG6000, the expression level of *KvHSP26* increased quickly after 2 hours, then slightly decreased after 6 hours and increased again after 12 hours (Figures 3(a) and 3(c)).

When treated with low temperature, the expression of *KvHSP26* decreased after 2 hours and then gradually increased back to the original level after 24 hours (Figure 3(b)). When treated with 100 *μ*M ABA, the expression level of *KvHSP26* increased after 6 hours and reached its highest level after 24 hours (Figure 3(d)). Compared to osmotic stress treatment with PEG, the response of *KvHSP26* to ABA treatment was a little delayed, but the induction was more significant.

## 4. Discussion

As a group of 12 to 42 kDa chaperone family proteins, the involvement of sHSPs in abiotic stresses has been well studied in glycophyte plants such as Arabidopsis [43], soybean [44], rice [45], wheat [46], poplar [47], pepper [48], cotton [49], willow [50], apple [23], and grape [51]. We isolated a *KvHSP26* gene from the halophyte plant *K. virginica*. Similar to the other reported *sHSPs*, *KvHSP26* encoded a 26.09 kDa protein with very high aliphatic index, a typical feature of heat shock proteins for their thermostability (Figure 1(a)). Based on their subcellular localization, sHSPs were divided into eleven subfamilies [16]. KvHSP26 showed high homology with other chloroplastic sHSPs from different plant species, with a 49 amino acid chloroplast transfer peptide in its N-terminal (Figure 1(b)). It is well known that the variable N-terminal of sHSPs contains the transiting, leading, or signaling sequence, which is responsible for their different subcellular localizations, whereas the conserved C-terminal contains the ACD domain, which is responsible for their proper biological functions [7, 17]. In *Malus sieversii*, a total number of 12 *HSP20* genes with 136-243 amino acids in length have been identified [23]. Our phylogenic and multiple sequence alignment analyses revealed that KvHSP26 showed high homology with other orthologous and paralogous genes from different plant species in close and distant evolutionary groups, containing a variable N-terminal and a conserved C-terminal ACD domain (Figures 1(c) and 1(d)). Since sHSPs could bind to the denatured proteins to help the successful binding of other chaperones, the N-terminal sequence diversity of sHSPs may reflect their substrate specificity [20, 21].

Various expression patterns of sHSPs have been observed in different plants. In willow tree, *sHsps* were specifically expressed in stems [50]. In potato, some *sHSPs* were ubiquitously expressed, whereas others were tissue specifically expressed [52]. In pepper, *CaHsp23.8* was constitutively expressed in stems, leaves, and flowers [48]. We found that *KvHSP26* was constitutively expressed in all tested tissues, with a higher expression in leaves (Figure 2(b)). Therefore, *KvHSP26* was a ubiquitously expressed gene in *K. virginica*.

Despite the various expression patterns of *sHSPs*, their participations in response to abiotic stress have been well studied. In potato, *sHSPs* responded to heat, salt, and drought stresses [52]. In wheat, *TaHSP16.9* expression was upregulated under salt stress condition [53]. In apple tree, the small heat protein gene *MsHsp16.9* was found to be involved in both ABA-dependent and ABA-independent signaling pathways [23]. We found that expression of *KvHSP26* increased in a concentration-/time-dependent manner under salt stress condition. With the increase of NaCl concentration or extension of treatment time, the expression level of *KvHSP26* increased correspondingly, suggesting its possible role in the salt tolerance of *K. virginica* (Figures 2(c) and 2(d)).

Previous studies have showed that expression of most *sHSPs* could be induced by unfavorable environmental conditions. The expression of small heat shock protein gene *CaHSP16.4* was strongly induced by both heat and drought stresses in pepper [24]. Recently, another sHSP in pepper, *CaHSP25.9*, was also identified to play important roles in the tolerance to heat, drought, and salt stresses [12]. The small heat shock protein gene *MsHSP17.7* of alfalfa was found to be induced by heat, NaCl, and osmotic stresses [33]. Consistent with these *sHSPs*, *KvHSP26* expression was also induced by heat, osmotic stress, and the phytohormone ABA in *K. virginica*. Upon the treatment of high temperature, the expression level of *KvHSP26* increased more than one thousand times after 2 hours (Figure 3(a)). So the *KvHSP26* gene could play a pivotal role in the heat response of *K. virginica*. Compared to high temperature, *KvHSP26* expression under low temperature decreased after 2 hours, then increased after 12 hours and recovered back to the original level after 24 hours (Figure 3(b)). The expression of *KvHSP26* was also induced by PEG, indicating its participation in drought response as well (Figure 3(c)). Additionally, the expression level of *KvHSP26* augmented remarkably upon ABA treatment. Therefore, *KvHSP26* may play a role in the responses to abiotic stresses via the ABA-dependent signaling pathway in *K. virginica*.

## 5. Conclusions

In this research, we isolated and characterized a new small heat shock protein gene *KvHSP26* from the halophyte plant *K. virginica*. Bioinformatics assays indicated that *KvHSP26* encodes a typical chloroplastic small heat protein with conserved sHSP (*α*-crystallin) domain in the C-terminal. qPCR analyses revealed that it was ubiquitously expressed with a higher expression in leaves and was dramatically induced by salt, heat, osmotic stress, and ABA. Our findings suggest that *KvHSP26* could play important roles in the tolerance to different abiotic stresses in *K. virginica*, and it has a great potential to be used as a candidate gene for the breeding of plants with improved tolerance to various abiotic stresses.

## Figures and Tables

**Table 1:** Primers used in this research.

Gene name	Application	Nucleotide sequence (5′-3′)	Tm (°C)	Size of product (bp)
*Hsp26-5-GSP*	RACE	F: CCTTGGCCTTGATTATCCTTG	49	331
		R: CGACGATTGGACTAATGGGAACT	53	
*Hsp26-3-GSP*	RACE	F: GTGCCCTTACGACCGACAGC	56	237
		R: CTAAGACCAAAGTGGAACGCAAAG	53	
*KvHSP26*	RACE	F: ATGGCAATGGCTTCAACTCTG	50	699
		R: TTACTGAATTTGTACATCAATAAC	45	
*KvHSP26-qRCR*	qPCR	F: CGACGATTGGACTAATGGGAACT	53	178
		R: CTTTGCGTTCCACTTTGGTCTTAG	53	
*Kv18SrRNA*	qPCR	F: CCGTTCTTAGTTGGTGGA	46	168
		R: AACATCTAAGGGCATCACAG	46	
*KvEF1-α*	qPCR	F: TCAATGAGCCAAAGAGG	44	185
		R: CAACACGACCAACAGGA	46	

## Data Availability

The data used to support the findings of this study are included within the article.
